# Assessing palliative care needs in Swabia: a data-driven simulation framework for hospice and specialized outpatient palliative care demand

**DOI:** 10.1186/s12904-026-02016-0

**Published:** 2026-02-18

**Authors:** Sara Garber, Eckhard Eichner, Stephanie Ludwig, Werner Schneider, Jens O. Brunner, Christina C. Bartenschlager

**Affiliations:** 1 Department of Statistics and Data Science, Faculty of Business and Economics, University of Augsburg, Augsburg, Germany; 2 Centre for Interdisciplinary Health Research, University of Augsburg, Augsburg, Germany; 3 Augsburger Palliativversorgung gemeinnützige GmbH, Augsburg, Germany; 4 St. Vinzenz-Hospiz Augsburg e. V., Augsburg, Germany; 5 Professor of Sociology with Consideration of Social Studies, Faculty of Philosophy and Social Sciences, University of Augsburg, Augsburg, Germany; 6 Faculty of Business and Economics, Faculty of Medicine, University of Augsburg, Augsburg, Germany; 7 Department of Technology, Management, and Economics, Technical University of Denmark, Lyngby, Denmark; 8 Center for Excellence in Healthcare Operations Planning, Next Generation Technology, Slagelse Hospital, Region Zealand, Denmark; 9 Professor of Applied Data Science in Health Care, Nuremberg School of Health, Ohm University of Applied Sciences Nuremberg, Nuremberg, Germany; 10 Clinic for Anaesthesiology and Operative Intensive Care, University Hospital of Augsburg, Augsburg, Germany

**Keywords:** Critically ill patients, Decision support in healthcare, End-of-life care, Prescriptive analytics, Statistics

## Abstract

**Background:**

The impact of demographic change has far-reaching consequences for the entire healthcare system. One of the areas particularly affected is palliative and hospice care. To meet the demand for palliative and hospice care services, adequate capacity planning in inpatient and outpatient settings is essential. However, the available capacities per one million inhabitants in Germany vary significantly within the individual regions.

**Methods:**

For an improved assessment of care needs, we propose a simulation-based analysis, carried out both retrospectively for 2019 and prospectively for 2039 for the southern German region of Swabia, and calculate the required capacities of hospices and specialized outpatient palliative care. We focus on cancer patients, as this group represents the largest proportion of patients requiring palliative and hospice care.

**Results:**

Driven by demographic change and the increasing incidence of cancer in older age groups, the number of patients requiring palliative care is expected to increase substantially. Accordingly, our results indicate a marked rise in the demand for hospice and specialized outpatient palliative care for cancer patients up to the year 2039. This projected trend underscores the growing care needs within this patient population in the coming decades.

**Conclusion:**

Our study introduces a simulation-based framework for estimating demand for hospice and specialized outpatient palliative care. By offering a quantitative, mathematical-statistical perspective, the approach complements existing qualitative research and supports informed decision making in palliative care planning.

**Supplementary Information:**

The online version contains supplementary material available at 10.1186/s12904-026-02016-0.

## Background

The impact of demographic change on the healthcare system will continue to increase in the coming years. This is partly driven by the growing demand for medical care, as the likelihood of developing, e.g., chronic illnesses steadily increases with age [1]. Palliative care aims to improve the quality of life of patients with serious and life-limiting illnesses, their families, and their caregivers [2]. Importantly, palliative care is not limited to end-of-life care but addresses the complex needs of patients across different stages of disease [2–5]. As such, it is one of the areas particularly affected.

Palliative care services encompass a range of organizational models that differ in structure, goals, and expertise. Specialist palliative care in Europe is delivered through multiple complementary service delivery models, including outpatient palliative care clinics, inpatient consultation teams, acute palliative care units, community-based palliative care, and hospice care [6]. Inpatient palliative care units provide coordinated and multidisciplinary holistic management as well as care orientation for patients with complex, life-limiting illness [7], whereas hospice care focuses primarily on comfort and support at the end of life. Furthermore, specialist palliative care is typically provided by multidisciplinary teams with specific training and expertise, offering intensive support for patients with high symptom burden and complex psychosocial needs [8]. Generalist or primary palliative care, on the other hand, is delivered as part of routine care by primary clinicians and non-specialists and addresses broader palliative needs at earlier stages of illness.

The urgency of planning for the increasing demand for end-of-life care and an aging population is critical not only in Germany but across Europe [9]. Therefore, proactive planning is essential to ensure that adequate capacity is available to meet the growing demand for end-of-life care. In this context, accurately estimating demand is crucial to effectively managing capacity in palliative and hospice care facilities. In the international literature, some papers address the development of methods for assessing the demand for palliative care among patients who require or could benefit from it, based on population [10–12]. In addition, several studies are investigating the application of methods for retrospective [13–19] and prospective [20–23] demand analyses for palliative care in different regions. All prospective studies illustrate the influence of demographic change on palliative care and show that many countries will face the challenge of a growing demand in the future [20–23]. The existing studies provide a comprehensive overview of the palliative care needs within specific populations, primarily focusing on estimating the number or proportion of affected individuals or deaths. However, these studies do not emphasize the impact of this demand on the capacity of care facilities, such as hospices and specialized outpatient palliative care (SOPC) institutions. As a result, interactions between projected demand and available care capacities remain insufficiently explored. In contrast, this study adopts an integrative approach by also considering the effects of the projected demand on the capacities of care facilities. In general, simulation-based approaches represent valuable decision-support tools for capacity planning and resource management in healthcare systems [24, 25] and can offer a nuanced understanding of future care needs and infrastructure requirements.

In addition to international literature, several papers examine the need for hospice and palliative care in various regions of Germany [26–38]. However, these analyses primarily rely on qualitative assessments. Furthermore, the available capacities in German hospices vary significantly across federal states [39]. In the Bavarian region of Swabia, there were 36 adult hospice beds available in 2019, equating to 19 per 1 million inhabitants. Additionally, 58 inpatient palliative care beds were operational that year, corresponding to 30.7 palliative care beds per 1 million inhabitants. In total, 49.7 stationary hospice and palliative care beds per 1 million inhabitants were available in Swabia in 2019 to treat critically ill patients. Furthermore, ambulatory care can be provided through SOPC. However, to the best of our knowledge, no data on the total treatment capacity for SOPC in Swabia is available. According to recommendations from the European Association for Palliative Care (EAPC) and the German Association for Palliative Medicine (DGP), there should be 40 to 50 hospice beds per 1 million inhabitants [39]. Overall, the EAPC recommended a total of 80 to 100 beds per 1 million inhabitants for hospice and palliative care [40]. Given the increasing number of patients in hospice and palliative care, including a growing number with non-cancer-related conditions, it is important to examine how these needs might be quantified. Simulation offers a suitable methodological approach to bridge the gap between demand estimation and capacity planning in palliative care systems.

Therefore, this study contributes to addressing the research gap regarding the quantification of needs in hospice and palliative care. The objectives are to develop and apply a novel simulation-based framework to quantify the demand for palliative and hospice care in Swabia, both retrospectively for 2019 and prospectively up to 2039. Thereby, we focus on critically ill cancer patients, as they are the largest group of patients requiring palliative and hospice care in this region. Specifically, the study aims to estimate current and future demand for hospice and SOPC as well as to assess the potential implications of rising demand on capacity planning and resource allocation within specialist services. By providing a data-driven, quantitative perspective, this work seeks to inform future planning, decision-making, and research in palliative care services.

## Methods

### Dataset

The study was based on real-world data from Augsburg Hospiz- und Palliativversorgung e. V. and St. Vinzenz-Hospiz Augsburg e. V., consisting of an anonymized dataset of 683 patients who started care at a facility in Augsburg in 2019. This year was selected to avoid potential bias in the results due to the COVID-19 pandemic. For each patient, the following characteristics were recorded: age, illness, treatment facility, admission date, discharge date, and discharge reason (i.e., due to death or completion of treatment at the respective facility). The primary focus of this study was on cancer patients, as they represented the largest group in hospice and palliative care facilities in Swabia. Additionally, specialized care services for children are not considered, as there are fundamental differences compared to adult care.

Data from the cancer registry of the Robert Koch-Institute on disease ($\:e$) and mortality ($\:m$) in Germany over a five-year period were utilized to estimate the care demand [41]. The five most recent years available online at the time of the data query, which were unaffected by the COVID-19 pandemic, were used: 2014 to 2018 for disease incidence and 2015 to 2019 for mortality data. The population was divided into different genders and age groups (0–44, 45–54, 55–64, 65–74, and ≥ 75). Based on this segmentation, the minimum ($\:a$), maximum ($\:b$), and average incidence ($\:c$) of disease and mortality were calculated for each age group and gender over the selected five-year period. Accordingly, the population in Swabia was segmented into different groups based on the demographic data from the associated districts and independent cities [42]. To estimate the total demand twenty years into the future, a population forecast for Germany in 2039 was applied to the Swabian population. This forecast assumed that factors such as birth and immigration rates remain constant in the chosen model [43]. Therefore, the focus was on changes in population structure driven by societal aging. This approach enabled a comparison of current capacity needs with projected changes in demand over the next 20 years for the care facilities considered.

### Simulation model and evaluation

For the simulation framework in Python, the simulation runs ($\:r\in\:R$), the population groups ($\:i\in\:I$), the time points considered ($\:t\in\:T$)^1^, the care facilities included ($\:v\in\:V$), and the proportion of patients to be cared for ($\:o$) were predetermined. The parameter *o* was initially set at 10%, which aligns with the SOPC guidelines for the Swabian region. Additionally, the probability of receiving treatment at a specific facility was calculated from the dataset proportions ($\:{h}_{1}$, $\:{h}_{2}$). We also account for requests from patients whose treatment did not occur in a hospice or through SOPC.

After initialization, the probability of a cancer disease ($\:{p}_{i}$) and the probability that palliative and hospice care is required due to the disease ($\:{q}_{i}$)^2^ ​were generated for each population group based on a triangular distribution, using the disease incidence and mortality rates in Germany (formulas (1) and (2); all index sets, parameters and mathematical formulas used in the simulation framework are provided in Appendix 8.1 and 8.2, respectively). A triangular distribution was selected for its simplicity, making it easy for physicians and decision-makers to understand and interpret in practical applications.

**Fig. 1:**
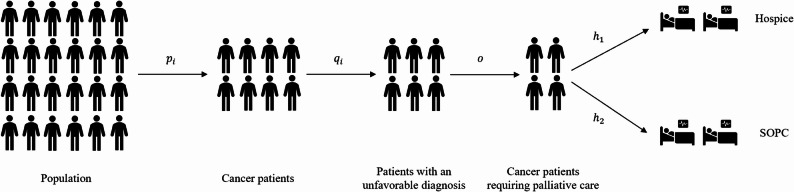
Graphical representation for calculating the number of patients to be treated in the hospice and SOPC care facilities (example)

Afterward, the number of patients to be treated per care facility ($\:{S}_{v}$) were calculated based on the number of individuals in each of the population groups in Swabia ($\:{N}_{i}$)^3^​ and the time period considered ($\:Z$) in the simulation (formula (3)). Overall, the proportion of cancer patients and those with an unfavorable diagnosis was based on population data related to disease incidence and mortality. The parameter $\:o$ was used to estimate the number of patients to be cared for by hospice or SOPC care, with subsequent capacity allocation determined by the proportions in the dataset ($\:{h}_{1}$, $\:{h}_{2}$). Fig. 1 provides an overview of the calculation of the number of patients to be treated in each care facility under consideration.

Subsequently, for each patient, a treatment start date within the considered time period, a treatment duration, and a treatment end date were assigned. The generation of the treatment start date was based on a uniform distribution, meaning each week could be assigned as the treatment start date for a patient with equal probability (formula (4)). In contrast, the treatment duration followed a triangular distribution, using the minimum ($\:{a}_{v}$), the maximum ($\:{b}_{v}$​), and the average treatment duration ($\:{c}_{v}$​), which depend on the care facility (formula (5)). Consequently, the treatment end date for each patient could be determined (formula (6)). The statistical key metrics of the distributions were derived from the minimum, average, and maximum treatment durations in the respective care facilities based on the dataset. It was assumed that the minimum treatment duration is one week and that a patient can only be treated for a whole number of weeks.

**Fig. 2:**
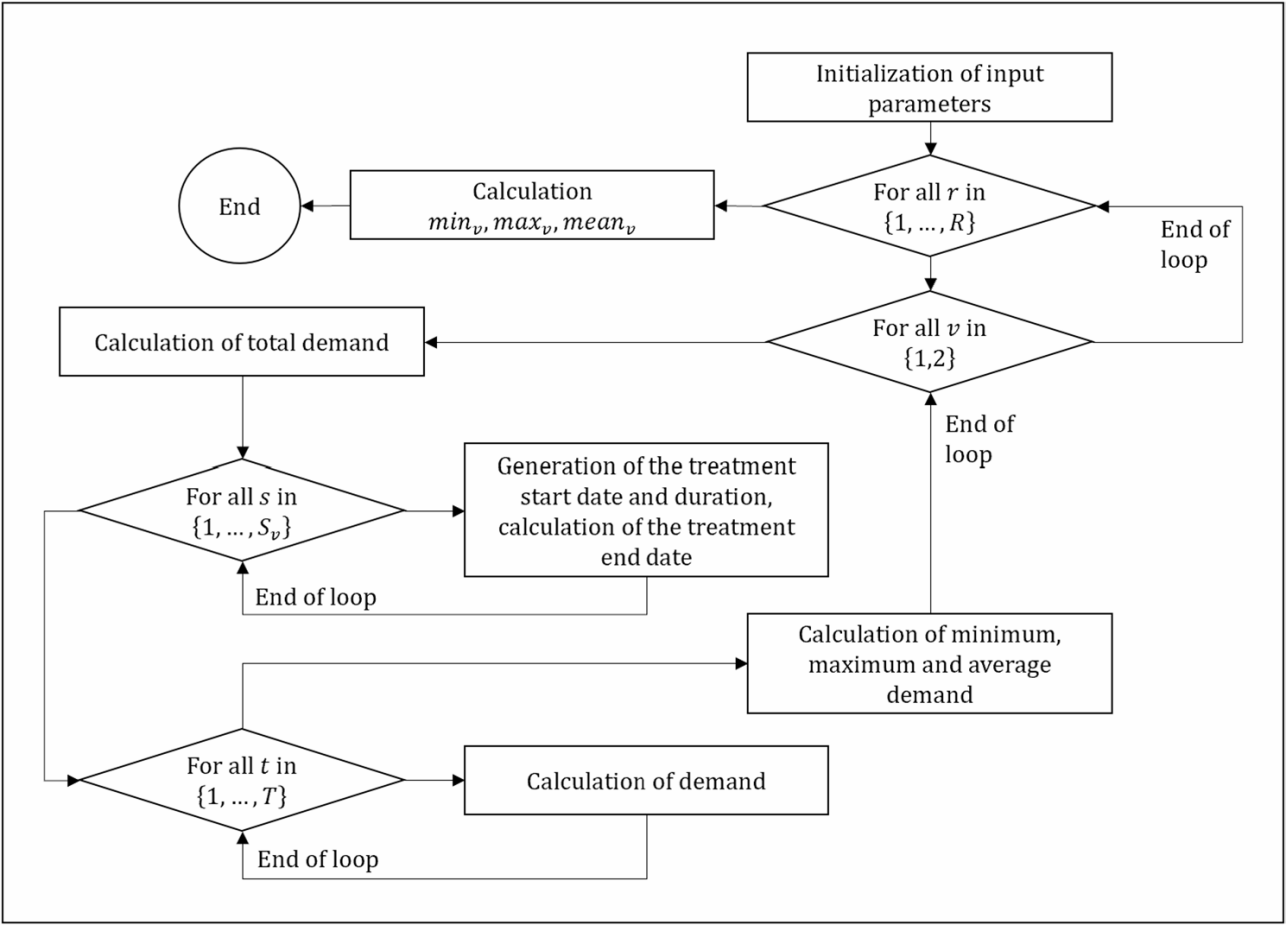
Flowchart illustrating the simulation setup in Python (Indices: $\:r$ – simulation run, $\:t$ – time point, $\:v$ – care facility, $\:s$ – patient)

Additionally, a binary variable indicated whether a patient’s treatment at a specific care facility occurred during the considered period (formulas (7) and (8)). Subsequently, for each considered time point (i.e., week within the five-year period), the demand for patients requiring care in each facility could be determined using formula (9). From these demands, the mean values of the minimum, maximum, and average demand across all simulation runs for each care facility could be calculated (formulas (10), (11), and (12)). Since the parameter $\:o$ representing the proportion of critically ill patients to be cared for was considered uncertain, a sensitivity analysis for the year 2019 was additionally conducted. A flowchart of the simulation framework, implemented in Python, is presented in Fig. 2. The calculations were performed for both 2019 and 2039.

## Results

### Data analysis

Out of the 683 patients considered, 172 were cared for in a hospice, while 511 received care through SOPC services. Among the patients analyzed, 513 (75.11%) were receiving treatment for cancer. The incidences of disease and mortality presented in Supplementary Table 1 in Appendix 8.3 are used to estimate the number of individuals diagnosed with cancer and in need of palliative and hospice care due to the disease. In addition, the percentage change in the Swabian population was calculated. While there are minimal changes in the age groups 0–44 years and 45–54 years for both genders, there is a noticeable decrease in the population aged 55–64 years and a significant increase in the age groups 65–74 years and ≥ 75 years (see Table 1). These changes are particularly relevant for the calculations, as the simulation framework focuses on the anticipated alterations in population structure driven by demographic shifts.

**Table 1:** Percentage change in the population in Swabia from 2019 to 2039, categorized by age group and gender. $\triangle_M$ represents the change in the male population, while $\triangle_W$ represents the change in the female population

Age group [years]	$\:{\varDelta\:}_{M}$	$\:{\varDelta\:}_{W}$
0–44	- 0,36 %	+ 1,47 %
45–54	- 3,64 %	- 5,51 %
55–64	- 19.03 %	- 17,96 %
65–74	+ 20,65 %	+ 18,90 %
$\:\ge\:$75	+ 24,44 %	+ 16,66 %

Furthermore, the demographic characteristics of patients at the care facilities in Swabia were subsequently analyzed. Over 90% of patients treated in 2019 fall within the age groups experiencing the most significant changes, i.e., 55–64 years, 65–74 years, and ≥ 75 years, highlighting the anticipated impact of demographic shifts in the future. In contrast, only a few patients from the other age groups received treatment. Table 2 presents the demographic characteristics of the patient cohort.

**Table 2:** Demographic data of the patient cohort considered

Age group [years]	Hospice	SOPC
0–44	1.16%	1.96%
45–54	7.56%	1.96%
55–64	18.60%	10.37%
65–74	28.49%	19.57%
$\:\ge\:$75	44.19%	66.14%

Additionally, the dataset analysis revealed notable differences in treatment durations between the two care facilities. Patients in the hospice had shorter lengths of stay (mean: 4 weeks) than those in SOPC (mean: 6 weeks). This variation highlights the differing care approaches and patient needs across settings. Supplementary Table 2 in Appendix 8.3 provides a detailed summary, presenting the minimum, maximum, and average treatment durations for both facilities.

### Simulation framework

In the simulation framework, the required capacities for cancer patients in hospices and SOPC facilities in Swabia were calculated. The simulation runs determined an average of 38 required hospice beds, corresponding to 20.1 beds per 1 million inhabitants, and 303 patients who needed to be cared for by SOPC simultaneously in 2019. In 2039, the simulation runs indicate an 11.11% increase in the average number of required hospice beds, to a total of 43 beds, along with a 12.35% increase in required SOPC capacity, to a total of 382 patients. A significantly higher demand could be observed across all statistical key metrics for 2039 (see Table 3).

**Table 3:** Statistical key metrics of the required capacities for the years 2019 and 2039 ($\:{min}_{v}$ – minimum, $\:{max}_{v}$ – maximum, $\:{mean}_{v}$ – mean) and the percentage change $\triangle_V$ in demand for the care facilities

Statistical key metrics	Hospice/SOPC (2019)	Hospice/SOPC (2039)	$\:{\varDelta\:}_{v}$
$\:{min}_{v}$	24/256	28/289	+ 16,67 %/ + 12,89 %
$\:{max}_{v}$	54/340	60/382	+ 11,11 %/ + 12,35 %
$\:{mean}_{v}$	38/303	43/343	+ 13,16 %/ + 13,20 %

**Fig. 3:**
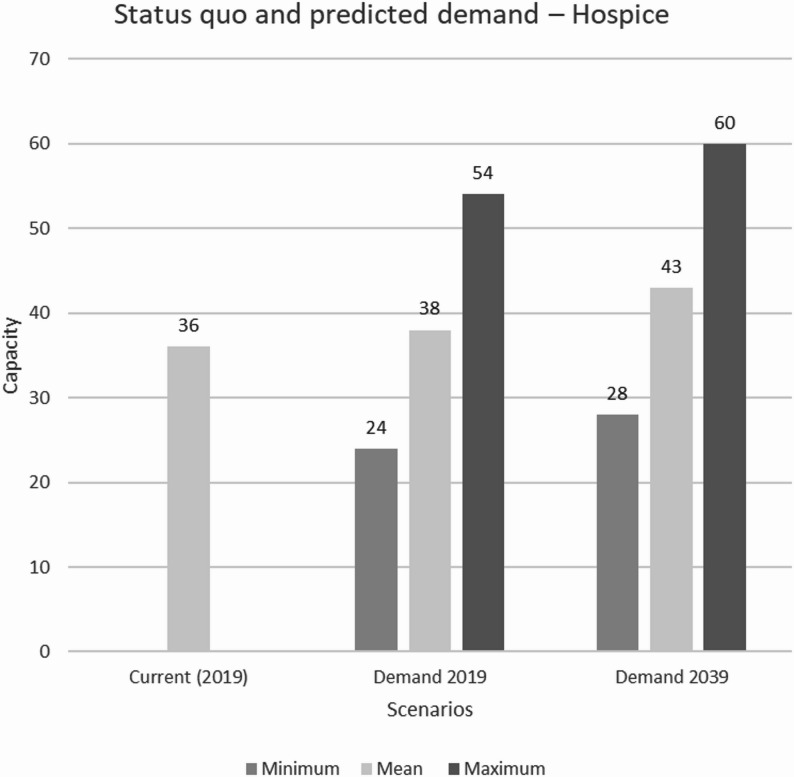
Comparison of the status quo and the simulated demand for hospice beds per week

**Fig. 4:**
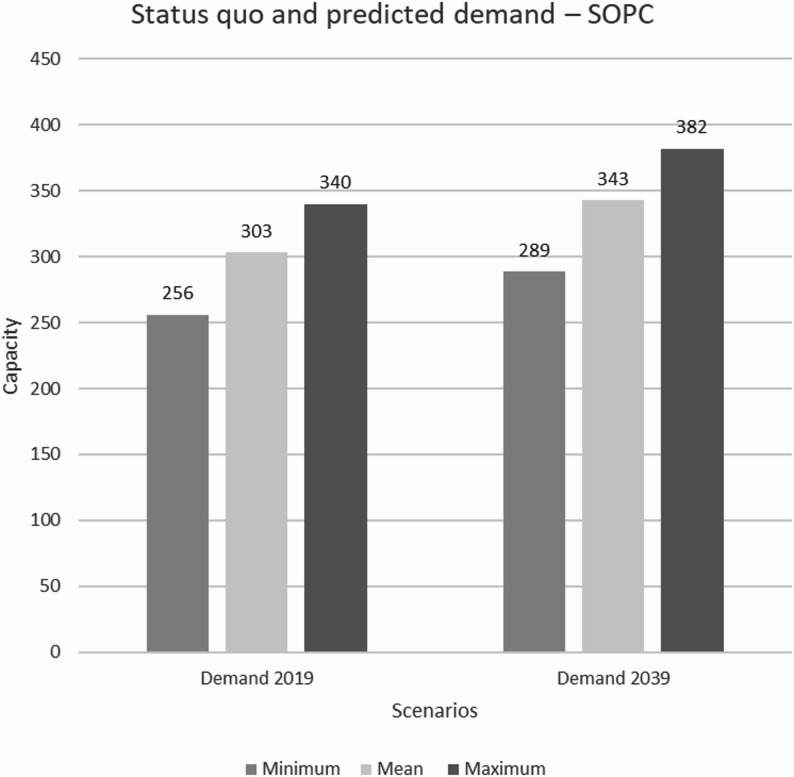
Comparison of the simulated demand for care provided by SOPC per week

The statistical key metrics were compared with the capacities in Swabia in 2019, accommodating a maximum of 36 patients in inpatient hospices. Figs. 3 and 4 provide a graphical representation. Additionally, the 10th and 90th percentiles of the mean were calculated to represent the range of average demand in both facilities, indicating that 80% of the values fall between these two points. For the hospice, the 10th percentile is 37 beds, and the 90th percentile is 39 beds in 2019, while for the SOPC, these percentiles correspond to 297 and 310 patients, respectively.

In 2039, the 10th and 90th simulated percentiles were 42 and 44 for the hospice, and 337 and 350 for the SOPC, respectively. For further evaluation, a sensitivity analysis was conducted for the parameter $\:o$, which determines the proportion of patients to be cared for, and is considered (very) uncertain and dynamic. For instance, if the parameter $\:o$ is set to 0.3, the average utilization corresponds to 115 beds in hospice care and 912 patients receiving SOPC treatment simultaneously. The minimum, maximum, and average required capacities, calculated based on variations in the patient proportion, are displayed graphically in Appendix 8.4 (Supplementary Figs. 1 and 2). In addition, a depiction of the distributions and frequencies of the averages across the simulation runs for each facility when using $\:o=0.1$ is included in Appendix 8.4 (Supplementary Figs. 3 and 4).

## Discussion

We used a simulation framework to estimate hospice and palliative care demand in Swabia based on demographic data and predefined assumptions about patient groups. In the framework, the number of hospice beds in Swabia in 2019 appeared to be insufficient to meet the needs of cancer patients at that time. While the expected low to moderate demand in the simulation could be met, more beds were needed during periods of increased demand (54 patients on average). The simulation for 2039 indicates that the gap between available and required capacity will widen, potentially leaving more cancer patients unable to secure a hospice place, even during periods of moderate demand. A comparison of the simulated demand with the number of patients treated by SOPC was not included due to insufficient data from individual facilities in Swabia. However, based on the results, it is expected that the number of patients requiring care at SOPC facilities will far exceed available capacity. The results suggest that the capacities in Swabia in 2019 will be insufficient in 2039 to meet even the demand of cancer patients. To address anticipated demand, the number of hospice beds in Swabia has already been expanded to 48 as of 2024. According to current plans, 58 to 66 beds are expected to be available in the medium term.

Overall, our study represents an initial exploration into assessing hospice and palliative care demand using quantitative methodology. It is important to note that this simulation framework focuses solely on cancer patients. As a result, it does not capture the full extent of actual demand, which is likely to be higher, particularly because the demand for non-cancer patients will become increasingly important in the future [26]. However, notable differences exist between the facilities in Swabia. The SOPC is experiencing a growing number of patients with internal or neurological conditions. This shift towards non-cancer populations has been increasingly described in literature and highlights the changing case mix in palliative care settings [13, 44–46]. In contrast, more than 90% of the patients receiving treatment in the hospice considered in this study are suffering from cancer disease. These differences already highlight the inherent challenges in accurately estimating demand in this field.

Furthermore, additional factors in the simulation framework contribute to an underestimation of the actual demand. For example, when calculating the number of cases, the incidence, i.e., the number of newly diagnosed patients, is used, which focuses on the needs of this specific patient group. If prevalence were used to account for all patients, including those already receiving care, the demand would be significantly higher. We also assume that 10% of critically ill patients will require care through hospice and SOPC. It is worth discussing whether this percentage, derived from SOPC guidelines, remains sufficient, especially given that Scholten et al. estimate that 40.7–96.1% of dying patients could benefit from palliative and hospice care [19]. In the sensitivity analysis of this critical parameter, a significant increase in demand is observed even with a relatively small increase. Overall, it is likely that the needs of cancer patients are underestimated in this study.

Given the increasing need for hospice and palliative care due to demographic change and the growing importance of other patient groups, it is unclear whether the recommendations of the EAPC and the DGP will meet future requirements in Swabia. As a result, long waiting times are to be expected. When patients cannot be accommodated, they are placed on a waiting list. Given the patient’s specific need, the following scenarios may occur: (1) death on the waiting list before possible admission; (2) deterioration of the patient’s condition requiring transfer to another care facility (e.g., from home to a hospital); (3) waiting for an available care place (if possible); or (4) patient stabilization removing the need for a care place. The specific outcomes depend on the patient’s location, and other scenarios may also arise.

An important contextual factor when interpreting and transferring the findings is the heterogeneity of health system organization and financing across European countries. European health systems differ substantially, ranging from tax-funded Beveridge models to social insurance-based Bismarck systems, as well as hybrid or transitional models [47]. These structural differences influence the organization, financing, and accessibility of palliative care services, potentially affecting capacity planning and service utilization. The present study is situated within a Bismarck-type health system, as in Germany, and the results should therefore be interpreted within this context. Applying the proposed framework to other health system settings may require adaptations to account for different financing mechanisms, referral pathways, and organizational structures of palliative care services.

Moreover, clinical complexity, rather than diagnosis alone, is a key determinant of palliative care needs. High symptom burden, psychosocial distress, multimorbidity, and care coordination challenges are central drivers for referral to specialist palliative care services, while patients with lower or fluctuating levels of complexity may be adequately supported within generalist or intermittent models of care [6, 40]. Importantly, palliative care needs are often dynamic rather than continuous. Some patients may require specialist input intermittently during periods of heightened symptom burden or transition, followed by phases in which care can be managed by generalist providers. Different models of specialized palliative care coexist and may have distinct implications for efficiency and patient outcomes. Community-based palliative care teams, in particular, have demonstrated favorable outcomes in symptom control, quality of life, and reduced hospital utilization [48], provided adequate caregiver support is available. These models may offer flexible and resource-efficient alternatives to inpatient care and should be further considered in future planning approaches.

An additional important consideration when interpreting the results of this study is the reliance on high-quality local data to inform the simulation parameters. While this approach strengthens the internal validity of the model, it may limit the transferability of the findings to settings with incomplete data, underreporting, or less robust information systems, where parameter estimates may become unstable or biased. Consequently, caution is warranted when applying or extrapolating the proposed framework to data-scarce contexts. Future applications could benefit from additional sensitivity analyses and local validation to better assess the robustness of the results under varying data quality conditions.

This study provides an initial step towards calculating hospice and palliative care demand using a quantitative, data-driven simulation framework. The challenges discussed in selecting assumptions and parameters required for such a quantitative analysis underscore the inherent complexity of this research question. In our view, it is essential to develop further and refine the framework to ensure the provision of care for critically ill patients even during periods of peak demand. Future research could enhance the methodology by incorporating a broader range of variables and refining assumptions to improve the precision of demand estimates. The continuing development of models and the collection of more robust data are critical to ensuring that capacities align with the evolving needs of the population.

## Limitations

This study is subject to several limitations. First, multiple factors may contribute to an underestimation of demand, as discussed in detail in the previous section. These include structural and organizational aspects that are not fully captured in the simulation framework. Nevertheless, this study provides the first quantitative approach to estimating the demand for palliative and hospice care services.

Second, the simulation framework simplifies real-world care dynamics. For example, it does not account for waiting lists, temporal fluctuations in demand, or overlaps between alternative or substitute care options, such as palliative care beds, SOPC, or hospice services in neighboring regions. Consequently, demand is smoothed over time, and the maximum number of patients receiving SOPC may vary depending on the required level of care. In addition, detailed and comparable data on staffing levels, particularly at the level of individual facilities in Swabia, are not available and are subject to considerable temporal variation, which precludes their explicit inclusion in the model.

Third, external influences and long-term developments are not explicitly modeled. In particular, the simulation does not account for demand fluctuations caused by external shocks, such as the COVID-19 pandemic. These events may have affected preventive screenings, early detection, and treatment pathways, potentially increasing future demand for palliative and hospice care services [49]. Although the use of pre-pandemic data was intentionally chosen to avoid short-term distortions caused by the COVID-19 pandemic, this decision may introduce other limitations. The pandemic has had profound and lasting effects on healthcare systems, which are not captured in our simulation. Consequently, demand patterns observed in recent years may differ from those modeled in this study. However, by relying on pre-pandemic data, our analysis aims to reflect structural demand under stable system conditions rather than crisis-driven fluctuations. Future extensions of the simulation framework could explicitly incorporate pandemic-related system changes, workforce constraints, and evolving referral criteria to better reflect current and future service dynamics. In addition, demographic changes beyond the year 2039 are not considered, although these may be relevant for long-term planning.

Fourth, the framework relies on high-quality local data to inform model parameters. While this strengthens the internal validity of the estimates, it limits the transferability of the results to settings with incomplete data, underreporting, or less robust information systems. Furthermore, regional demand estimates cannot be generalized to the whole of Germany due to substantial regional differences [39].

Finally, the simulation assumes full access to palliative and hospice care services within the study region. In practice, geographical, logistical, and organizational barriers may restrict access for some populations, an aspect that is not explicitly incorporated into the model. Nonetheless, our calculations offer a valuable starting point for future analyses and recommendations.

## Conclusion

This study provides a novel simulation-based demand analysis of palliative and hospice care in Swabia, both retrospectively for 2019 and prospectively for 2039. The simulation framework highlights the complexity of estimating the need for palliative and hospice care, underscoring the critical importance of further research to quantify demand.

Our simulation highlights a substantial increase in the demand for hospice and SOPC over the coming years. Furthermore, given the rising demand in practice, it is unclear whether the current recommendations of professional associations will remain sufficient to ensure adequate care. These findings underscore not only the need for adequate capacity planning within specialist services, but also the importance of strengthening palliative care competencies across all medical specialties. Incorporating a comprehensive approach in which generalist clinicians are trained in core palliative skills could improve early symptom management, psychosocial support, and timely referrals to specialist services. Such an approach may also help alleviate peak demand in the modeled network and enhance the overall responsiveness of the system.

In future research, our data-driven simulation framework for planning and managing outpatient and inpatient palliative care capacities should be further developed, with a robust data foundation grounded in standards. Additionally, the results should be examined and evaluated from a practical, qualitative, and interdisciplinary perspective.

## Supplementary Information

Supplementary Material 1.

## Data Availability

Due to data protection regulations, the dataset on hospice and SOPC patient characteristics used in this study is not publicly accessible. Please contact the corresponding author. The additional demographic data used in the study are freely available from the sources cited.

## Abbreviations

SOPC: Specialized outpatient palliative care
EAPC: European Association for Palliative Care
DGP: German Association for Palliative Medicine/Deutsche Gesellschaft für Palliativmedizin

## Footnotes

1 In the simulation, an artificial period before t=1 was generated for the assignment of treatment start dates to prevent distortion of the results. This period is not further considered and has no impact on the model.

2
$\:{q}_{i}$ represents the probability that a patient diagnosed with cancer will require care in a hospice or through SOPC due to this illness.

3
$\:{N}_{i}$​ represents the population size of the $\:i$-th demographic group.
