# Supplementary material for: Assessing palliative care needs in Swabia: a data-driven simulation framework for hospice and specialized outpatient palliative care demand
Source: BMC Palliat Care. 2026 Feb 18;25:55. doi: 10.1186/s12904-026-02016-0 (PMC12930724; doi:10.1186/s12904-026-02016-0)
Supplement: Supplementary file 1 — Supplementary Material 1. [file 12904_2026_2016_MOESM1_ESM.pdf]

8 Appendix

8.1 Index sets and parameters

<u>Index sets</u>

$R \coloneqq \{r \in N \mid 1 \leq r \leq 1000\}$

$I \coloneqq \{i \in N \mid 1 \leq i \leq 10\}$

$T \coloneqq \{t \in N \mid 1 \leq t \leq 261\}$

$V \coloneqq \{v \in N \mid 1 \leq i \leq 2\}$

$S_v \coloneqq \{ s \in N \mid 1 \leq s \leq |S_v| \}, v \in V$

<u>Parameters</u>

$O \coloneqq \{o \in R, o = 0.05 \cdot k, k \in \{1,2, \dots ,10\}\}$

$H \coloneqq \{h_v \mid h_1 = 0.27, h_2 = 0.73\}$

$A_e \coloneqq \{ a_i^e \mid i \in I \}$

$B_e \coloneqq \{ b_i^e \mid i \in I \}$

$C_e \coloneqq \{ c_i^e \mid i \in I \}$

$A_m \coloneqq \{ a_i^m \mid i \in I \}$

$B_m \coloneqq \{ b_i^m \mid i \in I \}$

$C_m \coloneqq \{ c_i^m \mid i \in I \}$

$A_v \coloneqq \{ a_v \mid v \in V \}$

$B_v \coloneqq \{ b_v \mid v \in V \}$

$C_v \coloneqq \{ c_v \mid v \in V \}$

$Z \coloneqq 5$

8.2 Mathematical model

$$p_i \sim D\left(\frac{a_i^e}{100{,}000}, \frac{b_i^e}{100{,}000}, \frac{c_i^e}{100{,}000}\right) \quad \forall i \in I \tag{1}$$

$$q_i \sim D\left(\frac{a_i^m}{100{,}000}, \frac{b_i^m}{100{,}000}, \frac{c_i^m}{100{,}000}\right) \quad \forall i \in I \tag{2}$$

$$S_v = \sum_{i \in I} N_i \cdot p_i \cdot q_i \cdot o \cdot Z \cdot h_v \quad \forall v \in V \tag{3}$$

$$e_{r,v,s} \sim U(1, T) \quad \forall r \in R, v \in V, s \in S_v \tag{4}$$

$$l_{r,v,s} \sim D(a_v, b_v, c_v) \quad \forall r \in R, v \in V, s \in S_v \tag{5}$$

$$d_{r,v,s} = e_{r,v,s} + l_{r,v,s} - 1 \quad \forall r \in R, v \in V, s \in S_v \tag{6}$$

$$x_{r,v,s,t} = \begin{cases} 1 & \text{if the treatment of } s \text{ occurs at } t \text{ and } v \text{ during } r \\ 0 & \text{else} \end{cases} \tag{7}$$

$$\sum_{t=e_{r,v,s}}^{d_{r,v,s}} x_{r,v,s,t} = l_{r,v,s} \quad \forall r \in R, v \in V, s \in S_v \tag{8}$$

$$y_{r,v,t} = \sum_{s \in S_v} x_{r,v,s,t} \quad \forall r \in R, v \in V, t \in T \tag{9}$$

$$min_v = \frac{1}{R} \cdot \sum_{r \in R} min_{t \in T}\, y_{r,v,t} \quad \forall v \in V \tag{10}$$

$$max_v = \frac{1}{R} \cdot \sum_{r \in R} max_{t \in T}\, y_{r,v,t} \quad \forall v \in V \tag{11}$$

$$mean_v = \frac{1}{R \cdot T} \cdot \sum_{r \in R} \sum_{t \in T} y_{r,v,t} \quad \forall v \in V \tag{12}$$

8.3 Tables

Supplementary Table 1: Incidences of disease and mortality of cancer patients in Germany, a_i indicates the minimum, b_i the maximum and c_i the mean value per 100,000 inhabitants.

Age group [years]	Statistical key metrics	Disease (male)	Disease (female)	Mortality (male)	Mortality (female)
0-44	a_i	51	76	7	9
	b_i	53	79	8	9
	c_i	52	78	8	9
45-54	a_i	314	471	86	89
	b_i	342	488	100	98
	c_i	324	480	94	94
55-64	a_i	963	771	329	244
	b_i	1015	816	376	259
	c_i	987	795	354	254
65-74	a_i	2002	1192	816	514
	b_i	2028	1230	852	523
	c_i	2012	1216	833	519
≥75	a_i	2649	1581	1880	1105
	b_i	2790	1658	1962	1140
	c_i	2727	1631	1916	1119

Supplementary Table 2: Statistical key metrics for the duration of treatment in care facility v are represented as follows: a_v indicates the minimum, b_v the maximum, and c_v the mean value.

Duration of treatment	Hospice	SOPC
a_v	1	1
b_v	38	115
c_v	4	6

8.4 Figures

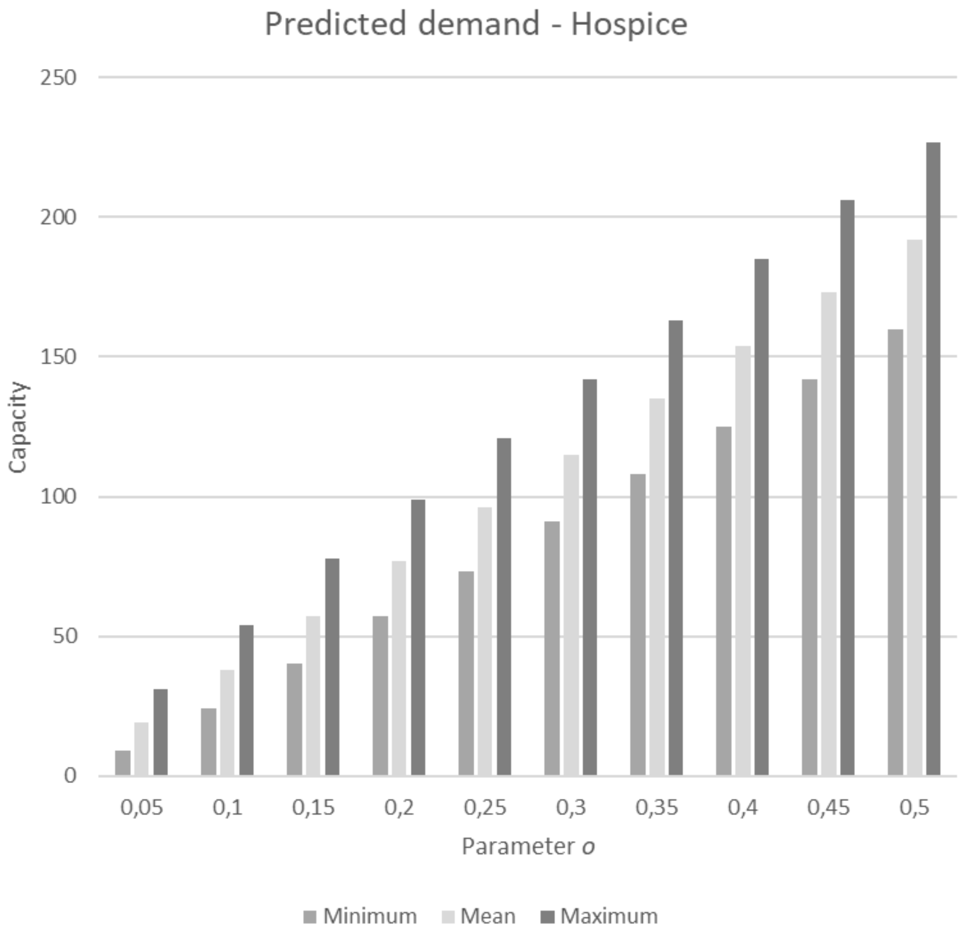

Supplementary Figure 1: Sensitivity analysis of the simulated demand for hospice beds per week (year 2019).

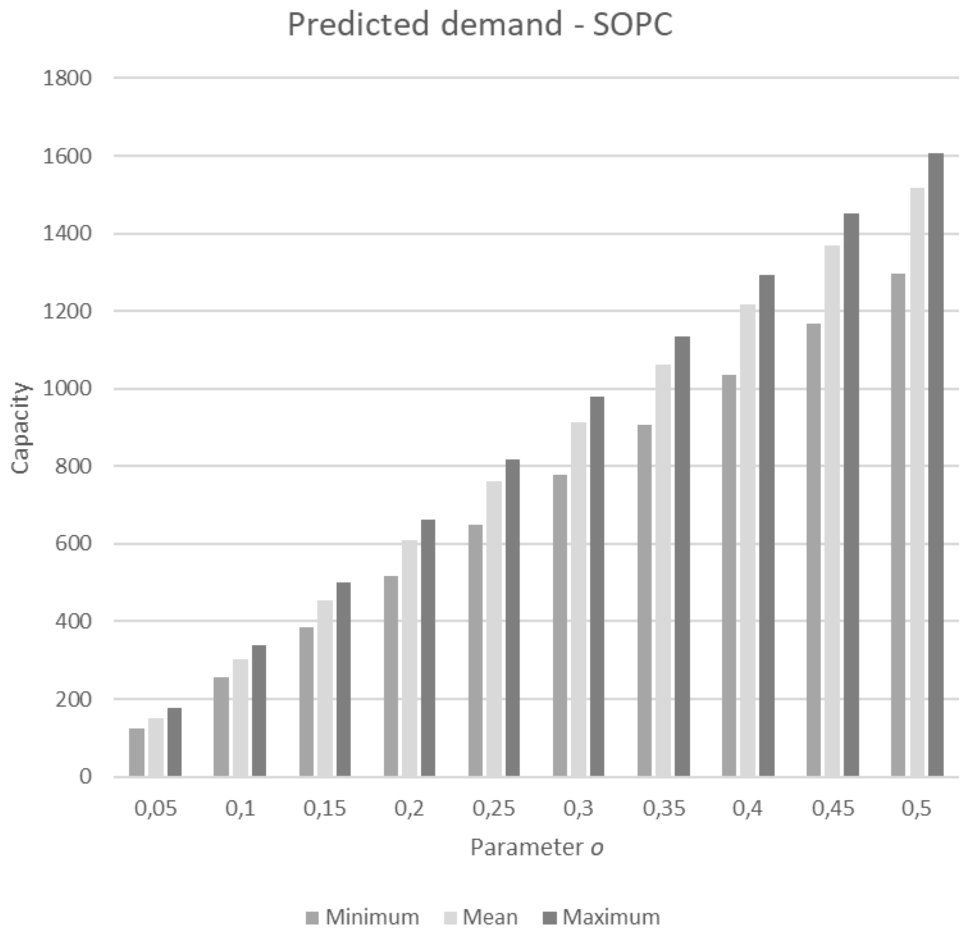

Supplementary Figure 2: Sensitivity analysis of the simulated demand for care provided by SOPC per week (year 2019).

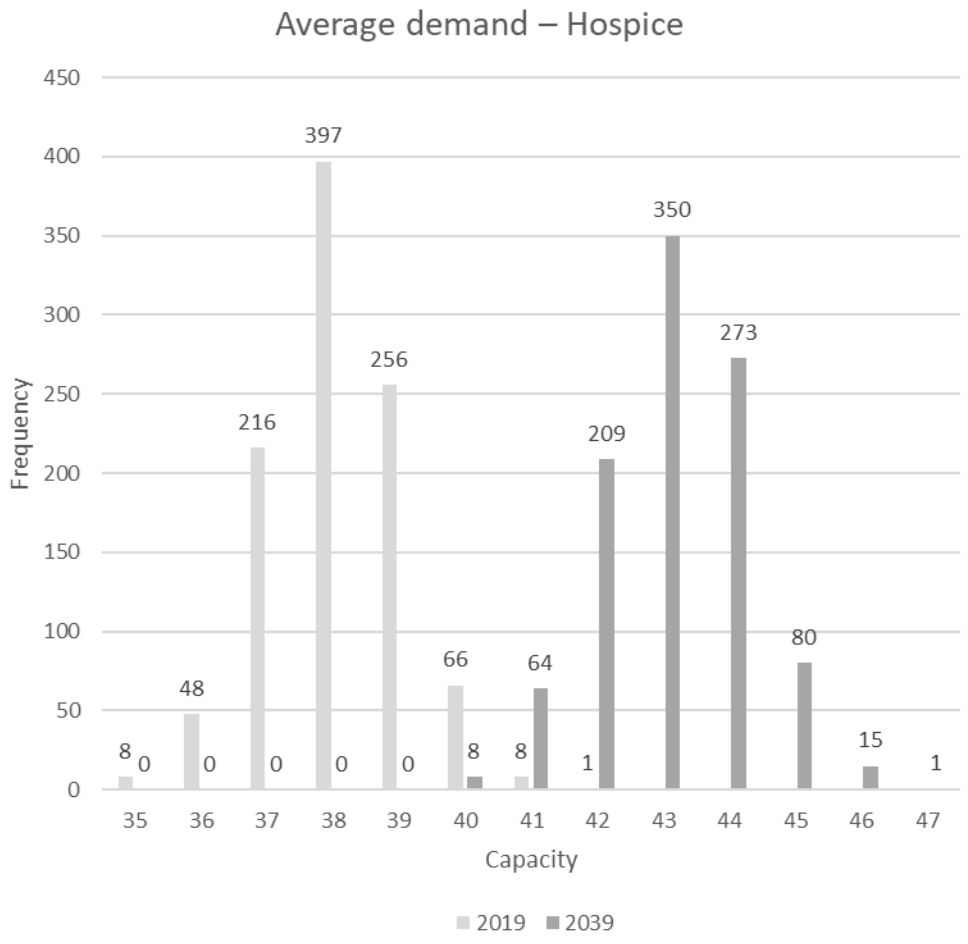

Supplementary Figure 3: Average required hospice capacities for 2019 and 2039, along with their frequency in the simulation runs.

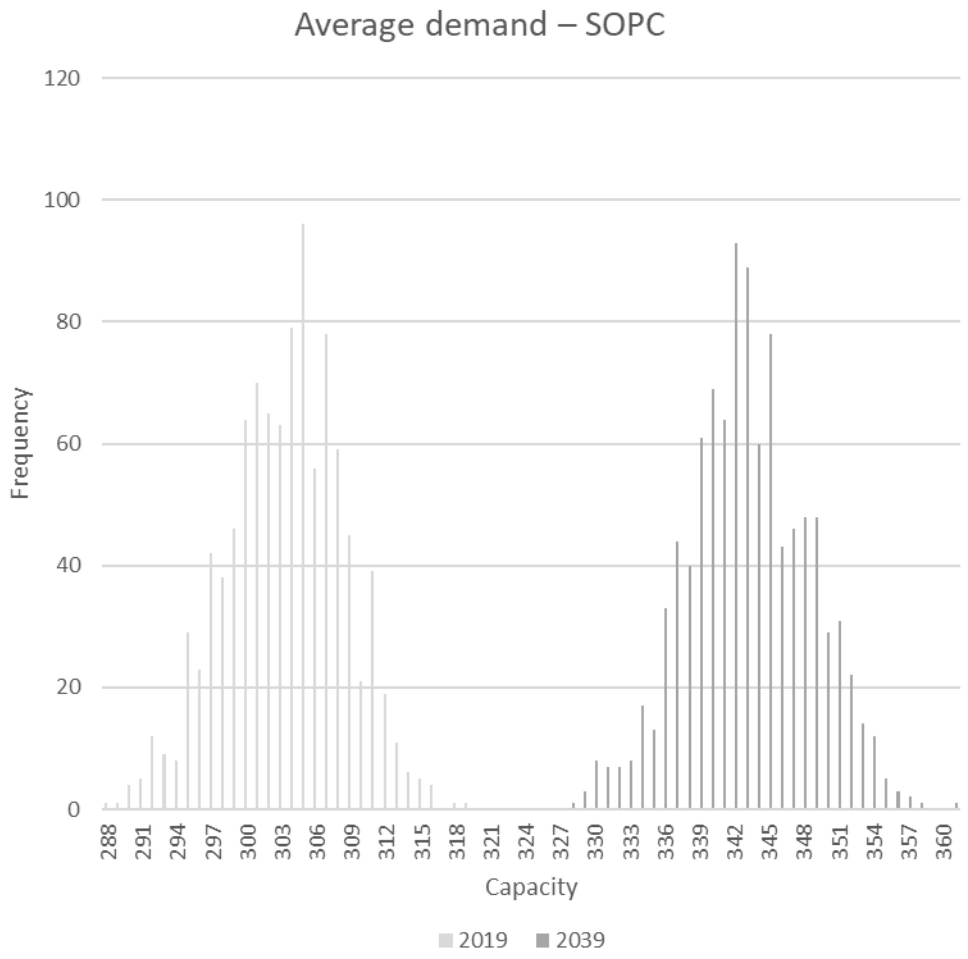

Supplementary Figure 4: Average required SOPC capacities for 2019 and 2039, along with their frequency in the simulation runs.
